# Multidimensional and Longitudinal Approaches in Talent Identification and Development in Racket Sports: A Systematic Review

**DOI:** 10.1186/s40798-023-00669-2

**Published:** 2024-01-07

**Authors:** Sebastiaan B. Nijenhuis, Till Koopmann, Jesper Mulder, Marije T. Elferink-Gemser, Irene R. Faber

**Affiliations:** 1 Department of Human Movement Sciences, University of Groningen, Groningen, The Netherlands; 2 Institute of Sport Science, Carl Von Ossietzky University Oldenburg, Ammerländer Heerstraße 114-118, 26129 Oldenburg, Germany; 3 Research Centre Human Movement and Education, Windesheim University of Applied Sciences, Zwolle, The Netherlands; 4 Department of Public Health and Primary Care, Leiden University Medical Center/Health, Campus The Hague, Leiden, The Netherlands

**Keywords:** Racquet sports, Aptitude, Talent, Youth sports, Sports performance

## Abstract

**Background:**

Better methods to support talent identification and development processes may contribute to more effective and efficient athlete development programs in racket sports. Both researchers and practitioners recommend multidimensional and longitudinal approaches to better understand the nature of talent (development). However, the added value of these ‘innovative’ approaches has not yet been clarified for racket sports. This systematic review intends to gain further insight into the outcomes of multidimensional and longitudinal approaches for talent identification and development in racket sports and to provide directions for future talent research.

**Methods:**

Electronic searches were conducted in PubMed, Scopus, SPORTDiscus, and Web of Science (January 2000–August 2022). Search terms covered the areas of racket sports and talent in sports. Studies using multidimensional and/or longitudinal talent approaches were included and analyzed regarding the methodology, included performance characteristics (i.e., anthropometrical, physiological, technical, tactical, psychological), and study findings.

**Results:**

A total of thirty-two studies were included using multidimensional (*n* = 15), unidimensional longitudinal (*n* = 3) or multidimensional longitudinal designs (*n* = 14). Most research covered physiological characteristics (*n* = 28), while fewer articles investigated anthropometrics (*n* = 21) and technical characteristics (*n* = 16). Only limited research investigated psychological (*n* = 4) and tactical characteristics (*n* = 1). Almost all studies measured physiological characteristics in combination with other characteristics. There was moderate to strong evidence that physiological and technical characteristics have value for athlete development programs in racket sports. Positive but limited evidence was found for psychological and tactical characteristics. Anthropometrical assessments were generally used as controlling variables for maturation. Study designs varied, and many studies used unidimensional statistical models and analyses within multidimensional study designs and datasets.

**Conclusions:**

This review provides an overview of talent research using multidimensional and/or longitudinal approaches within racket sports and gives guidance on what characteristics to include in decision-making and monitoring processes. However, it remains difficult to draw conclusions about the added values of these approaches due to their variety in methodology. Future talent research should include more consistent study designs and conduct multidimensional and longitudinal studies using multivariate statistical approaches that benefit from the data’s multidimensionality.

**Supplementary Information:**

The online version contains supplementary material available at 10.1186/s40798-023-00669-2.

## Key Points

- Results show that specifically physiological and technical, but also psychological and tactical characteristics are valuable for talent identification and development in racket sports.
- The current evidence regarding the added value of multidimensional and longitudinal approaches in talent research in racket sports is limited and allows only preliminary conclusions.
- Future talent research should focus on more consistent study designs and conduct multidimensional as well as longitudinal studies including various characteristics and multivariate statistical approaches.

**Supplementary Information:**

The online version contains supplementary material available at 10.1186/s40798-023-00669-2.

## Background

An increasing number of talent identification and development (TID) programs are installed by sports associations to identify young athletes with the potential to achieve future success and provide them with the most optimal opportunities and environments to develop [1–3]. However, identifying talent and discovering how to optimally stimulate young athletes’ development remains a difficult challenge in most sports and contexts. Consequently, sports associations are searching for new solutions to increase a program’s efficacy and efficiency. One could argue that young athletes need to require a certain baseline performance/skill level to be able to compete and be eligible for a TID program in the first place. As such, programs include the development and implementation of assessments that measure certain individual characteristics (e.g., anthropometric, physiological, technical) suggested to influence or determine future performance. Unfortunately, it appears that most objective measures can only explain a small part of future performance. This might be due to the use of unidimensional and/or cross-sectional designs in most conventional TID studies. Such approaches seem to be oversimplifying the complexity and evolving nature of talent in sports [1]. It is uncontentious that athletic performance is multidimensional [1, 4] and that the developmental process is a highly individual and nonlinear pathway involving many interacting factors [3, 5–11]. As a response, scientists have been calling for studies using multidimensional and longitudinal approaches to better understand the complex nature of talent [12].

The Groninger Sports and Talent Model (GSTM) for the development of a talented athlete’s performance is in accordance with such an approach as it suggests defining performance in a multidimensional way and monitoring development over time [13, 14]. The model is based on Newell’s constraints-led model and defines sports performance as the result of the interaction between individual, task, and environmental characteristics [15]. In the GSTM, the individual characteristics describe factors that relate to the individual qualities of athletes, which are divided into five categories: anthropometrics, physiological, technical, tactical, and psychological characteristics. Task characteristics represent the sport and its specific demands, and environmental characteristics include the surroundings of the athlete, e.g., sociodemographic characteristics of the athlete. The interactions between the individual characteristics, the task and the environment change over time under the influence of individual maturation, learning, and training processes. It is of essence to reveal the ‘key’ characteristics related to performance and to obtain insight into these developmental processes as this helps to improve the development of young talented athletes toward expert performers. Here, a longitudinal approach is expected to be more informative than a cross-sectional approach [14, 16]. Longitudinal studies involve the repeated assessment of the same individuals over a longer time period (e.g., 6 months). Such studies have the advantage of capturing the long-term changes in athletes’ performance characteristics and relating them to future career-related outcomes to discover which characteristics could be important for successful future performance [2]. Furthermore, longitudinal data can contribute to the creation of realistic goals and training procedures in talent development [17]. For example, a study in handball (*n* = 94, age 13–17 years) revealed how multiple individual characteristics developed over time in different age- and performance groups [18]. This study showed the importance of specific performance-related characteristics and how the characteristics as well as their importance developed over time in this specific sport, helping coaches to evaluate players.

This review intends to gain further insight into the outcomes of not only longitudinal but also multidimensional approaches and to provide directions for future talent research by means of a focused study on individual characteristics in racket sports. Racket sports are generally considered early specialization sports (at 6–10 years of age), and peak performance is frequently reached relatively late (25–35 years of age) and can last a long period (up to 15 or 20 years) [19]. As such, players aiming for the elite level and other stakeholders connected to this developmental process (e.g., parents, trainers, coaches, clubs, and associations) need to invest great amounts of resources to increase the chances of successes. Better methods to support talent identification and development within racket sports are likely to contribute to more effective and efficient TID programs. This can be of great value for both players and other stakeholders when deciding about their pathway and investments. The assessment of individual characteristics and their relationship to performance have already been reviewed for racket sports [19–21]. For example, instruments focusing on intellectual and perceptual abilities and coordinative skills were able to discriminate between various performance levels. However, their predictive validity was not yet confirmed. Furthermore, there was moderate evidence that assessing mental and goal management skills could predict future performance [19]. Also, the assessments of sport-specific technical skills could discriminate different performance levels and predict future performance in TID activities in different sports [21]. Moreover, there was strong evidence that technical and tactical skills differentiate performance levels specifically in tennis [20]. Nevertheless, these reviews emphasized that the individual performance-related characteristics were mostly measured in isolation and/or on a single measurement occasion. This indicates that a limited number of previous studies used a multidimensional and/or longitudinal research design.

In the recent past, more and more multidimensional and longitudinal studies have been conducted. However, to the best of our knowledge, there is no comprehensive overview of empirical/data-driven multidimensional and/or longitudinal research in racket sports. Synthesizing the knowledge on talent development and the individual characteristics in racket sports specifically using multidimensional and/or longitudinal insights would allow for a better understanding of the developmental processes and help to identify and guide young talented players to using their full potential. Therefore, this systematic review aims to provide an overview of empirical/data-driven multidimensional and/or longitudinal research in talent development within the field of racket sports. It intends to gain further insight into the outcomes of multidimensional and longitudinal approaches for talent identification and development in racket sports and provide directions for future talent research by means of the following research question: Which (set of) individual performance-related characteristics can explain performance outcomes in young talented racket sport players? This work contributes to a better understanding of what can be learned from the current literature, identify gaps and provide possible directions for future research.

## Methods

### Search Strategy and Eligibility Criteria

The Preferred Reporting Items for Systematic Reviews and Meta-Analyses (PRISMA) guidelines were followed when conducting and reporting this review [22]. Electronic database searches were conducted in PubMed, Scopus, SPORTDiscus, and Web of Science [23]. The search was limited to peer-reviewed papers published in English from January 2000 until the 12th of August 2022. Search terms for all databases represented the following racket sports: tennis, table tennis, badminton, squash and padel. Additional search terms represented the concepts of talent, talent identification, and talent development as well as sports performance. Because the word “squash” has multiple meanings, some terms concerning fruit, vegetables, and plants were added to the search string using the operator NOT. In summary, the search for articles contained the following terms:

(“racquet sport* (MeSH)” OR “racket sport*” OR racquetball OR “racquet ball” OR racketball OR “racket ball” OR tennis (MeSH) OR “table tennis” OR squash OR badminton OR padel).

AND.

(Talent* OR aptitude*(MeSH) OR gift* OR assess* OR endowment* OR select* OR scout* OR expert* OR elite OR excellen* OR success* OR perform* OR identif* OR develop*).

NOT.

(pumpkin* OR intervention* OR vegetable* OR fruit* OR plant*).

Articles were included if they (1) were original articles containing an empirical/data-driven study using inferential statistics, (2) focused on the identification or development of talented young players in at least one of the major racket sports (i.e., tennis, table tennis, badminton, squash, and padel), and (3) included a multidimensional and/or longitudinal approach. Multidimensionality was met when at least two of the five individual characteristics (i.e., anthropometric, physiological, technical, tactical, and psychological) of the GSTM were covered in a study to compare athletes of different performance levels (e.g., elite versus sub-elite) and/or to explain performance (e.g., rating score or ranking). With this, the ‘psychological’ category covered both the psychological and cognitive performance determining factors [24] and the technical category included both technical skills (e.g., stroke velocity and accuracy) and (perceptual-)motor skills (e.g., eye-hand coordination). Study designs covering multiple measurements during a minimum time period of 6 months were defined as longitudinal studies. In both the multidimensional and longitudinal studies, the measure(s) had to be related to (any aspect) of talent or sport-specific performance outcomes. Exclusion criteria applied in this review were articles (1) concerning factors beyond the individual (i.e., task or environmental characteristics), (2) with insufficient relationship between individual characteristics and (a measure of) talent or performance (e.g., relationship between individual characteristics), (3) including solely group comparisons regarding sex (i.e., male versus female), age (i.e., younger versus older), maturation (e.g., early versus late maturing), handedness (i.e., left-handed versus right-handed), sports (e.g., racket sports versus swimming and judo) or countries (e.g., German versus Dutch athletes) without a clear performance outcome, (4) concerning the comparison of different experimental conditions or intervention studies, and (5) focusing on non-healthy or injured participants. Furthermore, duplicates and articles without full-text access were also excluded. Titles, abstracts, and full-text articles were screened by four authors (SN, TK, JM, IF) using the web tool “Rayyan” [25]. If judgment differed between the authors, articles were discussed within the research group until consensus was reached.

### Data Extraction/Synthesis

Study characteristics were manually extracted into custom Excel workbooks [26]. The dataset included the following information regarding the article: name of the authors, publication year, sport(s) investigated, sample’s country of origin, sex, size and age, study design reported, dimensions measured, measurements conducted, and general findings. Subsequently, the samples’ performance level was determined based on the method of Swann et al. (2015) [27]; samples were classified as semi-elite, competitive elite, successful elite, or world-class elite athletes.

### Quality of Evidence

The methodological quality of the articles included was evaluated using a modified checklist based on the Strengthening the Reporting of Observational Studies in Epidemiology (STROBE) Statement. Modifications were based on the adaptations by Koopmann et al. (2020) [21]. Articles were assessed based on a 16-item list including a study’s (1) title and abstract, (2) scientific background and rationale, (3) objectives and hypotheses, (4) information on data collection, (5a) participant information, (5b) participant selection, (6) outcome variables, (7) statistical methods, (8) missing data, (9) main results, (10) results reported in statistical terms, (11) sources of bias, (12) post hoc comparisons, (13) summary of key results, (14) limitations, (15) interpretation of results, and (16) generalizability. The outcome was reported by allocating “0” (does not fully meet criteria), “1” (meets criteria), or “NA” (not applicable). The methodological quality was independently assessed by two researchers (SN, WE) and discussed until consensus was reached. When there was doubt, the research group was consulted. A total score was calculated by summation of the scores on each item. Percentage scores were calculated as the final score, calculated by dividing the total score by the number of relevant scored items (i.e., NA items were not included). Articles were categorized as having a low, moderate, or high methodological quality based on the percentage ≤ 60%, 61–79%, and ≥ 80%, respectively. These thresholds are in line with the cut-off scores used by recent reviews in sports science [19, 21, 28]. Subsequently, information on the studies’ quality and their findings were combined to rate the level of evidence for the dimensions within different sports. The level of evidence was categorized as strong (i.e., three studies of high OR five studies of moderate quality with/without statistically significant effects), moderate (i.e., two studies of high OR three studies of moderate quality with/without statistically significant effects), limited (i.e., one study of high OR two studies of moderate quality with/without statistically significant effects) or conflicting (i.e., < 2:1 ratio between studies with and without statistically significant effects) [19, 21].

## Results

**Fig. 1:**
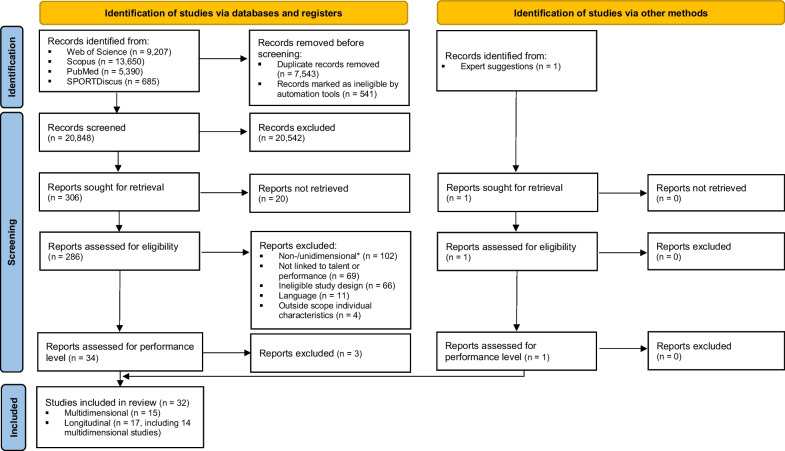
Flow chart systematic search. *If not longitudinal

The systematic search yielded 28,932 articles from all four databases (Fig. 1). After removing duplicates (*n* = 7543), excluding articles based on automatic eligibility (*n* = 541), title and abstract (*n* = 20,542), and irretrievability (*n* = 20), 286 articles remained for full-text screening. Subsequently, 252 articles were excluded because the study design was not multidimensional or longitudinal (*n* = 102), the article had an insufficient relationship to talent or high-performance (*n* = 69), the study design of the article was ineligible (*n* = 66), the article was not written in English (*n* = 11) or the article was outside the scope of the five individual characteristics (*n* = 4). Articles with only semi-elite athletes were also eliminated considering the insufficient representation of talented or high-performing athletes (*n* = 3). One additional article was proposed during the review process which met the inclusion criteria. Finally, 32 articles remained for inclusion and were of low (*n* = 1), moderate (*n* = 6) and high (*n* = 25) methodological quality. The included articles were labeled as multidimensional (*n* = 15) and/or longitudinal (*n* = 17, including 14 studies using a combined multidimensional and longitudinal approach) and comprised tennis (*n* = 19), table tennis (*n* = 7), badminton (*n* = 4), squash (*n* = 2), and padel (*n* = 1). The distribution of the individual characteristics taken into account within the multidimensional studies is presented in Additional file 1: Table S1 of the supplementary files. The characteristics of the multidimensional and longitudinal studies can be found in Tables 1 and 2, respectively. Table 3 presents the results of the methodological quality check for all articles. In the following sections, the studies’ findings regarding various characteristics are presented starting with the characteristic investigated the most and ending with the one studied the least.

**Table 1:** Multidimensional studies included in the systematic review

Author(s) (Year)	Sport *performance level*	Sex subgroup (sample size)	Reported age Mean age ± SD or range (years)	Reported study design	Statistical approach	Dimensions	Reported measurements	Findings
*Two-dimensional*								
Abdullahi et al. (2017) [37]	Badminton *competitive elite*	Female (9) Male (20) Total (29)	NR NR 21.24 ± 6.41	Prospective, descriptive, cross-sectional	Independent t-test^a^ (national vs. provincial)	Anthropometric Physiological	Weight, height, skinfolds, chest width, arm span SR, VJ, long jump, sit-ups, push-ups, sprint speed	National players have better motor fitness parameters. No differences were found for anthropometrics and flexibility
Baiget et al. (2014) [29]	Tennis *Successful elite*	Male Total (38)	18.2 ± 1.3	NR	Multiple regression model/analysis (competitive performance)	Physiological Technical	HR, VO2max, ventilatory thresholds Technical effectiveness	Low to moderate correlations were found between performance (final stage), physiological (VT1, VT2) and technical effectiveness, and competitive performance (*r* = 0.35–0.61; *p* = 0.038–0.000). Technical effectiveness explained 37% of variability in competitive performance (*r* = 0.61; *p* = 0.001). Using technical effectiveness combined with endurance measures or predictability increased explaining approximately 55% (*p* < 0.05) of the variance in competitive performance
Baiget et al. (2016) [30]	Tennis *Successful elite*	Male *International (8)* *National (30)* Total (38)	17.9 ± 1.0 18.3 ± 1.40 NR	Descriptive, correlational	T-test/Welch’s test Stepwise discriminant analysis (national vs. international)	Physiological Technical	HR, VO2max, ventilatory thresholds Technical effectiveness	Aerobic fitness and technical efficiency can discriminate between national and international tennis players. In the discriminant analysis, only technical efficiency variables are included and 86% of the players were classified correctly
Brechbuhl et al. (2018) [31]	Tennis *Successful elite*	Female *Junior (14)* *Senior (13)* Total (27)	14.7 ± 1.0 18.9 ± 3.2 16.7 ± 3.1	NR	One-way ANOVA^a^ (junior vs. professional) Pearson rank order correlation analysis	Physiological Technical	Time to exhaustion, (maximum) oxygen uptake, HR, blood lactate, ventilatory thresholds Technical performance (ball accuracy- and velocity)	Compared with juniors, professionals possess higher exercise capacity, maximal, and submaximal aerobic attributes along with faster backhand stroke velocities during an incremental tennis-specific field test
Catalán-Eslava et al. (2018) [40]	Squash *Competitive elite*	Male Total (80)	33.46 ± 8.24	NR	Mann–Whitney U test/Kruskal–Wallis^a^ (national vs. national vs. regional vs. provincial)	Technical/Tactical	Squash Performance Evaluation Tool (HERS); control, decision, and execution	National players show higher technical and tactical skill levels compared to regional and provincial players
Chen et al. (2022) [34]	Table tennis *Successful elite*	Male *Advanced (10)* *Intermediate (10)* Total (20)	20.6 ± 1.2 20.6 ± 1.5	NR	Independent t-test^a^ (advanced [division I] vs. intermediate [division II])	Physiological Technical	Kinematic joint angles, joint torques, EMG Racket speed	Advanced players can produce higher upper- and lower-limb joint angular velocities for faster racket speeds. Furthermore, they demonstrated longer firing duration and higher muscle force generation in some muscles Advanced players were capable to produce higher shoulder, hip, and knee torques at faster speeds, but not at lower speeds
Filipcic et al. (2015) [35]	Tennis *Competitive elite*	Female (NR ^b^) Male (NR^b^)	12–17 12–17	NR	MANOVA (tennis players vs. school pupils, 12/13 yrs vs. 14/15 yrs old vs. 16/17 yrs old, female vs. male)	Anthropometric Physiological	Height, weight, BMI Polygon, forward bend, hand-tapping, sit-ups	Tennis players were taller than average school pupils and outperformed them in all physiological measurements, across sex and age categories as well as measurement periods. Findings concerning weight and therefore also BMI were inconsistent
James et al. (2022) [38]	Squash *Competitive elite*	Male (21) Female (10) Total (31)	20 ± 4 18 ± 5 NR	NR	Two-way ANOVA^a^ (performance level [high world ranking vs. low world ranking] vs. sex [female vs. male])	Anthropometrics Physiological	Stature, body mass, skinfolds, girth measures Squash physical performance test (SPPT), mean submax oxygen consumption, VO2max, 5 m sprint, COD, repeated-sprint ability (RSA), SJ, CMJ, blood lactate	Higher-ranked players performed better for SPPT final lap and COD. Assessments of cardiovascular fitness, RSA, COD, and body composition appear highly pertinent for performance profiling of squash players
Kurtz et al. (2019) [32]	Tennis *Competitive elite*	Female (14) Male (15) Total (29)	18–25 18–25 18–25	NR	Multiple linear regression predicting Universal Tennis Ranking	Physiological Technical	Spider test, footwork taps Serve- forehand- and backhand velocity	Serve, forehand and backhand velocity, agility, and endurance explain 86.6% of the variance in tennis rankings. Also, all variables correlate strongly to rankings in collegiate athletes. Tennis-specific endurance correlates moderately to ranking
Sánchez-Muñoz et al. (2020) [39]	Padel *Successful elite*	Male *Elite (25)* *Sub-Elite (35)* Total (60)	31.1 ± 5.7 25.3 ± 5.9 27.7 ± 6.4	NR	Independent t-test^a^ (elite [PPT events] vs. sub-elite [(pre-)qualifying rounds])	Anthropometric Physiological	Stature, body mass, arm span, skinfolds, girths, breadths, width, and length of the hands, BMI, body fat%, muscle mass CMJ, grip strength, lumbar isometric strength, SR	Elite padel players had lower body fat and higher lumbar isometric strength than sub-elite players. No differences were found for the other measurements
Söğüt (2017) [33]	Tennis *Competitive elite*	Male (18) *Elite (8)* *Club (10)* Female (17) *Elite (7)* *Club (10)* Total (35)	13.43 ± 0.79 13.60 ± 0.70 11.88 ± 0.83 12.20 ± 1.32 NR	NR	Mann–Whitney *U*-test^a^	Physiological Technical	KTK Serve velocity	Elite players had higher scores for serve speed and motor coordination than club-level players
Ziemann et al. (2011) [36]	Tennis *Competitive elite*	Female Total (17)	15–17	NR	Pearson correlation^a^	Anthropometric Physiological	Height, weight, BMI, fat-free mass, body fat%, fat mass VO2max, Wingate anaerobic power test, blood lactate	Ranking position was not significantly correlated to body composition. Also, ranking position was significantly negatively correlated with VO2max (*r* = − .682, *p* < .01). Finally, ranking position was not significantly correlated with Wingate test results Ranking positions were related to aerobic capacity. Anaerobic capacity was related to BMI and lean body mass but was of minor importance for ranking positions
*Three-dimensional*								
Robertson et al. (2022) [43]	Badminton *Competitive elite*	Male *Elite (10)* *Sub-elite (24)* *Novice (27)* Total (61)	15.22 ± 1.33 15.41 ± 1.56 15.79 ± 1.89 NR	NR	MANCOVA and discriminant analysis	Anthropometrics Physiological Psychological	Body height, sitting height, body weight, fat%, BMI SR, knee push-ups, sit-ups, standing broad jump, shuttle run, 5-,10-, 20-, 30-m sprint, endurance shuttle run, CMJ with and without arms. Jumping sideways, moving sideways, balance beams (KTK) Psychological characteristics of developing excellence questionnaire version 2	Significant differences were found in physical performance (explosive power, flexibility, speed, and endurance), BMI, and motor coordination and elites scored highest. In the psychological domain, perfectionism was found to be significantly different and elites scored highest. The discriminant analysis, combining anthropometry, physical performance, motor coordination, and psychological traits, showed that 100% of the participants were correctly classified and 80.0% were correctly cross-validated
Sánchez-Pay et al. (2021) [41]	Tennis *World-class elite*	Male Total (15)	19.66 ± 1.63	NR	*T*-tests^a^ (professional vs. national)	Anthropometrics Physiological Technical	Body mass, body height, BMI, arm-, forearm-, thigh-, leg-length Grip strength, CMJ, MBT Serve velocity, jump on service	Professional level players showed higher values in all parameters (except in MBT shot put), although no statistically significant differences were found between level groups (*p* > .05)
Ulbricht et al. (2016) [42]	Tennis *Competitive elite*	Male (546) Female (366) Total (912)	13.14 ± 1.39 13.06 ± 1.29 NR	NR	Independent sample t-test^a^ for differences between national and regional players Spearman’s rank correlations for the relationship between performance variables	Anthropometrics Physiological Technical	Height, weight, sitting height Grip strength, CMJ, 5-, 10-, 20 m sprint, MBT, tennis-specific sprint test, hit and turn test Serve velocity	Results showed that serve velocity (*r* = 20.43–0.64 for female subjects [♀]; r = 20.33–0.49 for male subjects [♂]) and upper-body power (e.g., MBT *r* = 20.26–20.49 ♀; *r* = 20.20–20.49 ♂) were the most correlated predictors of tennis performance (i.e., national youth ranking) in both female and male tennis players. Moreover, national players showed better performance levels than their regional counterparts, mainly in the most predictive physical characteristics (i.e., serve velocity: effect size [ES], 0.78–1.04 ♀; ES 0.92–1.02 ♂, MBT: ES, 0.66–0.88 ♀; ES, 0.67–1.04 ♂) and specific endurance (ES, 0.05–0.95 ♀; ES, 0.31–0.73 ♂)

*BMI* body mass index, *CMJ* counter movement jump, *COD* change of direction, *HR* heart rate, *KTK* Körperkoordinationstest für kinder, *MBT* medicine ball throw, *NR* not reported, *SJ* Squat Jump, *SR* sit-and-reach, *VEmax* maximum minute ventilation, *VJ* Vertical Jump, *VO2max* maximum oxygen uptake

^a^Univariate analysis

^b^Sample size varied between measurements. Total number of unique players is not reported

**Table 2:** Longitudinal studies included in the systematic review

Author(s) (Year)	Sport *performance level*	Sex subgroup (sample size)	Reported age Mean age ± SD or range at first testing (years)	Testing timespan	Reported study design	Statistical approach	Dimensions	Reported measurements	Findings
*Unidimensional*									
Banzer et al. (2008) [44]	Tennis *World-Class* *elite*	Male (1)	NR	7 years	Prospective, case-report	Cross-correlation for pairwise comparison	Physiological	VO2max	A strong relationship was found between VO2max and the following year’s ATP entry ranking during 7 years of professional tennis
Gale-Watts & Nevill (2016) [46]	Tennis *World-Class elite*	Male (NR)	NR	29 years	NR	Nonlinear cubic polynomial regression model	Anthropometric	Height, Weight, BMI, reciprocal ponderal index (RPI)	Elite male tennis athletes are becoming more power athletes as opposed to endurance athletes given the increase in BMI and decrease in RPI for Grand Slam tournament participants
Martinent et al. (2018) [45]	Table tennis *Competitive elite*	Male (109) Female (50) Total (159)	NR NR 14.07 ± 2.13	6 years	NR	ANCOVA (active vs. dropout; international vs. national vs. regional)	Psychological	Sport motivation scale, coping inventory for competitive sport, athlete burnout questionnaire, recovery stress questionnaire	Results of ANCOVAs showed that players who still practiced at time 2 (T2; six years later; *n* = 130) reported lower time 1 (T1; while they were involved in intensive training centers) amotivation (large effect), disengagement-oriented coping, sport devaluation, and reduced accomplishment (moderate effects) than their counterparts who dropped out at T2 (*n* = 29). Results of ANCOVAs also showed that international (*n* = 18) and/or national players (*n* = 86) at T2 reported significantly lower T1 amotivation (large effect), disengagement-oriented coping, and sport devaluation (moderate effects) in comparison with regional (*n* = 26) players at T2. Finally, results of correlational analyses showed that T2 performance and/or 6-year performance progress were significantly and weakly correlated with introjected and external regulations, perceived stress, and perceived recovery, and significantly and moderately correlated with amotivation, disengagement-oriented coping, sport devaluation, and reduced accomplishment
*Two-dimensional*									
Faber et al. (2016) [53]	Table tennis *Competitive elite*	Male (24) Female (24) Total (48)	NR NR 7–11	2.5 years	Observational prospective	Generalized Estimating Equations analysis	Physiological Technical	Sprint, agility, VJ Speed while dribbling, aiming at target, ball skills, throwing a ball, eye-hand coordination	Perceptual-motor skills assessment outcomes do not predict competition participation Perceptual-motor skills assessment can objectify a young player’s potential when assessed at age 7–11 years. Yet, the Generalized Estimating Equations analysis, including the test items ‘aiming at target’, ‘throwing a ball’, and ‘eye-hand coordination’ in the best fitting model, revealed that the outcomes of the perceptual-motor skills assessment were significant predictors for future competition results (*R*^2^ = 51%)
Faber et al. (2017) [54]	Table tennis *Competitive elite*	Male (739) Female (452) Total (1191)	NR NR 7–10	15 years	Observational, test–retest	Univariable and multivariable logistic and linear regression models	Physiological Technical	Sprint, agility Speed while dribbling, throwing a ball	The test items “sprint” and “throwing a ball” showed to be significant predictors for table tennis performance outcomes in boys (*p* < 0.05). For girls, besides these test items also “speed while dribbling” had a significant contribution (*p* < 0.05). Since the accuracies of the models were low, it is advised to include other determinants to enhance the predictive value of a model for table tennis performance
Kanehisa et al. (2006) [47]	Tennis *Competitive elite*	Male		2 years	NR	Friedman test, Wilcoxon test, Mann–Whitney U test^a^ (table tennis players vs. non-athletes; compared only for strength test)	Anthropometric Physiological	Height, weight, thigh girth, cross-sectional area, skeletal age Dynamic Strength	The findings indicate that young tennis players who are in the earlier stage of adolescence increase the CSA of the quadriceps femoris muscle beyond the normally expected growth change. Also, they show a predominant development in torque generation capability during high-velocity knee extensions, with a greater gain in boys compared with girls
		*Elite (6)*	12.1 ± 0.5						
		*Control (29)*	11.5 – 14.4						
		Female							
		*Elite (6)*	12.0 ± 0.9						
		*Control (30)*	11.5 – 14.4						
		Total (71)	NR						
Kolman et al. (2021) [57]	Tennis *Competitive elite*	Male Total (29)	13.4 ± 0.51	4 years	Prospective	Multiple linear regression analysis (future elite vs. future competitive)	Anthropometric Technical	Height, weight Ball speed, accuracy, percentage errors	Ball speed and accuracy were significant predictors of current and future performance (*p* < .001) in male youth tennis players, with *R*2 of .595 and .463, respectively. When controlling for age, a one-way MANCOVA revealed that future male elite players were more accurate than future competitive players (*p* = .048, 95% CI [.000–.489]), especially in variable compared to fixed game situations (*p* < .05)
Kramer et al. (2016) [50]	Tennis *Competitive elite*	Male Total (256)	10–15	5 years	Mixed-longitudinal	Multilevel random effects regression analyses	Anthropometric Physiological	Height, weight CMJ, 5-m sprint	Players developed their 5-m sprint performance with age. The development is related to longitudinal changes in body size and lower-body power in elite young tennis athletes aged 10–15 years
Kramer et al. (2016) [48]	Tennis *Competitive elite*	Male (113) Female (83) Total (196)	NR NR 13–15	2 years	Mixed-longitudinal	Multilevel analysis (higher ranked vs. lower ranked)	Anthropometric Physiological	Height, weight SJ, CMJ, MBT, ball throwing, spider test, linear sprint tests	Physical fitness components for boys and girls improved over age (U14-U16) (ES .53–.97). In boys, the more mature boys outscored the less mature boys in upper and lower-body power from U14 to U16. In girls, high-ranked girls outscored lower-ranked girls on lower-body power, speed, and agility (U14–U16) (*p* < .05). In other words, boys and girls improved on all physical fitness components during U14–U16. In boys, power was related to maturity. In girls, lower-body power, speed, and agility were related to tennis performance
Kramer et al. (2017) [49]	Tennis *Competitive elite*	Male (44) Female (42) Total (86)	12.43 ± 0.30 12.48 ± 0.22 NR	3 years	NR	Regression analyses	Anthropometric Physiological	Height, Sitting height, weight, leg length MBT, ball throw, SJ, CMJ, 5,- 10-m sprint, spider test	At U13, maturation and physical fitness are partly related to tennis performance. In boys, higher scores on upper body power resulted in better tennis performance. However, none of the physical fitness tests at U13 were a predictor for tennis performance at U16 for boys
Kramer et al. (2021) [51]	Tennis *Competitive elite*	Female Total (167)	10–15	9 years	Mixed-longitudinal	Multilevel analysis	Anthropometric Physiological	Height, weight CMJ, 5-m sprint speed	It was possible to predict sprint performance (5 m) based on chronological age, body size given by height, and lower limb strength performance (*p* < .05). Significantly different developmental patterns were found for elite and sub-elite players, with elite players aged 10–14 being faster. After age 14, no significant differences were found in sprint performance between elite and sub-elite players (*p* > .05)
Madsen et al. (2018) [52]	Badminton *Competitive elite*	Male Total (10)	13.5 ± 0.5	2 years	Longitudinal	One-way ANOVA for repeated measures^a^ (test performance of age groups)	Anthropometric Physiological	Height, weight, fat%, arm span, thigh circumference 30-m sprint, CMJ, badminton-specific speed, badminton-specific endurance, HR	Athletes improve badminton-specific speed over time, achieving values at U19 similar to world-class elite senior athletes. At U19, there is still a performance deficit in badminton-specific endurance compared to world-class elite senior athletes
*Three-dimensional*									
Chapelle et al. (2022) [58]	Tennis *Competitive elite*	Male (323) Female (215) Total (538)	7–12	9 years	Cohort	Univariate binary logistic regressions^a^	Anthropometric Physiology Technical	Body height, body weight, sitting height, maturity offset 5 m sprint speed, 20 m sprint speed, 505 COD test, standing broad jump (in series), balancing backward, sideways jumping (KTK) Throw and catch, hold tennis ball up	Significant odds ratios were found for all included anthropometric and physical performance determinants (*p* < 0.05), ranging from 0.26 to 7.50 in the male young tennis players and from 0.18 to 6.87 in the female young tennis players. The included determinants influenced selection opportunities mostly in the early age categories (U8–U10) as opposed to the later age categories (U11–U13)
Siener & Hohmann (2019) [56]	Table tennis *Competitive elite*	Male (141) Female (84) Total (225)	94.1 ± 5.2 months	7 years	Prognostic validity	Linear discriminant analysis and neural network (multilayer perceptron) (talented [national level] vs. non-talented [club, local, regional level])	Anthropometric Physiological Technical	Height, weight, BMI 20-m sprint, sideward jumping, balancing backward, bend forward, push-ups and sit-ups, standing long jump, 6-min run Ball-throw	A medium to high prognostic validity could be proven with the complete motor test battery as well as with the table tennis recommendation score. For table tennis, six of the nine tests are recommended (sideward jumping, push-ups, bend forward, standing long jump, ball-throw, and balancing backward)
Siener et al. (2021) [59]	Tennis *Competitive elite*	Male (112) Female (62) Total (174)	156.3 (132–206) months	3 to 9 years	Retrospective (long-term)	MANCOVA, bivariate correlations	Anthropometric Physiological Technical	Height, weight 20-m sprint, sideward jumping, balancing backward, bend forward, push-ups, sit-ups, standing long jump, 6-min endurance run Ball-throw	No significant (*p* < 0.05) differences were found between ranked and non-ranked junior players in terms of U9 body weight and height. With the exception of flexibility, all physical fitness tests and motor competence tests showed significant results. The ball throw was the most relevant test parameter, as it showed the highest prognostic validity (effect size *ƞ*^2^ = .157 and *r* = .360). This test was followed by the two test tasks standing long jump (effect size *ƞ*^2^ = .081 and *r* = .287) and endurance run (effect size *ƞ*^2^ = .065 and *r* = .296)
Zhao et al. (2020) [60]	Mixed *Competitive elite*	Male *Table tennis (7)* *Badminton (4)* Total (21)	145.6 ± 8.0 months	2 years	Mixed cross-sectional and longitudinal	Discriminant analysis and a Neural Network (Multilayer Perceptron)	Anthropometric Physiological Psychological	Height, weight, chest girth Vital capacity, hemoglobin concentration, HR, back strength Eye-hand reaction time	Values in hemoglobin concentration, VC, body height, body weight, chest girth, and dynamic back strength increased over 2 years. The developmental pathways of anthropometric, physiological, and motor performance in elite Chinese athletes are not different from Caucasian athletes
*Four-dimensional*									
Doherty et al. (2018) [55]	Table tennis *Successful* *elite*	Male Total (14)	15.3 ± 1.2	1 year	Observational, prospective	Spearman’s rank-order correlation^a^	Anthropometric Physiological Technical Psychological	Height, weight, sitting height Sprint Eye-hand coordination Questionnaires: behavioral regulations, work engagement, cognitive emotion regulation, mental toughness, self-regulation of learning self-report	Significant correlations emerged between (a) actual performance rating and age from peak height velocity (*r* = .71), sprint test (*r* = − .69), number of years of practice (*r* = .84), positive refocusing (*r* = − .58), and self-regulation in learning—self-monitoring (*r* = − .60), and evaluation (*r* = .57); (b) performance rating one year later and positive refocusing (*r* = − .58), self-monitoring (*r* = − .50), and number of years of practice (*r* = .80). Results also showed significant correlations between progression scores (2017 rating score minus 2016 rating score) and age from peak height velocity (*r* = − 0.77), sprint test (*r* = .63), number of years of practice (*r* = − .52), self-monitoring (*r* = .69), and evaluation (*r* = − .58). Current performance correlated with sprint, training experience, positive refocusing, self-monitoring, and evaluation. Positive refocusing and training experience was able to predict future performance rating. Progression scores correlated with sprint, self-monitoring, and evaluation

*BMI* body mass index, *CMJ* counter movement jump, *COD* change of direction, *HR* heart rate, *KTK* Körperkoordinationstest für kinder, *MBT* medicine ball throw, *NR* not reported, *SJ* Squat Jump, *SR* sit-and-reach, *VEmax* maximum minute ventilation, *VJ* Vertical Jump, *VO2max* maximum oxygen uptake

^a^Univariate analysis

**Table 3:** Results of the quality check using a modified checklist based on the STROBE statement

Author(s) (year)	Abstract/Title	Introduction		Methods					Results				Discussion				Total	Total	Methodological quality
	#1	#2	#3	#4	#5a,b	#6	#7	#8	#9	#10	#11	#12	#13	#14	#15	#16	Score	%	
Abdullahi et al. (2017) [37]	1	1	1	1	(1,0) 0	1	1	0	1	0	0	1	1	0	1	0	10	63	Moderate
Baiget et al. (2014) [29]	1	1	1	1	(1,1) 1	1	1	0	1	0	1	NA	1	1	1	1	13	87	High
Baiget et al. (2016) [30]	1	1	1	1	(1,1) 1	1	1	0	1	0	1	1	1	1	1	0	13	81	High
Banzer et al. (2008) [44]	1	1	1	1	(0,0) 0	1	1	NA	1	0	0	NA	0	1	1	0	9	64	Moderate
Brechbuhl et al. (2018) [31]	1	1	1	1	(1,1) 1	1	1	0	1	1	1	1	1	1	1	0	14	88	High
Catalán-Eslava et al. (2018) [40]	1	1	1	1	(1,1) 1	1	1	0	1	0	0	1	1	1	1	1	13	81	High
Chapelle et al. (2022) [58]	1	1	1	1	(1,1) 1	1	1	0	1	1	0	NA	1	1	1	0	12	80	High
Chen et al. (2022) [34]	1	1	1	1	(1,1) 1	1	1	0	1	0	0	NA	1	1	1	1	12	80	High
Doherty et al. (2018) [55]	1	1	1	1	(1,1) 1	1	1	0	1	1	1	NA	1	1	1	1	14	93	High
Faber et al. (2016) [53]	1	1	1	1	(1,1) 1	1	1	1	1	1	1	NA	1	1	1	1	15	100	High
Faber et al. (2017) [54]	1	1	1	1	(1,1) 1	1	1	1	1	1	1	NA	1	1	1	1	15	100	High
Filipcic et al. (2015) [35]	1	1	1	1	(0,1) 0	1	1	0	1	0	1	1	0	0	1	0	10	63	Moderate
Gale-Watts and Nevill (2016) [46]	1	1	1	1	(1,1) 1	1	1	NA	1	0	1	NA	1	1	1	0	12	86	High
James et al. (2022) [38]	1	1	1	1	(1,1) 1	1	1	0	1	1	0	NA	1	1	1	0	12	80	High
Kanehisa et al. (2006) [47]	1	1	1	1	(1,1) 1	1	1	1	1	0	0	NA	1	1	1	1	13	87	High
Kolman et al. (2021) [57]	1	1	1	1	(1,1) 1	1	1	1	1	1	0	NA	1	1	1	1	14	93	High
Kramer et al. (2016) [50]	1	1	1	1	(1,1) 1	1	1	NA	1	1	0	NA	1	1	1	0	12	86	High
Kramer et al. (2016) [48]	1	1	1	1	(1,1) 1	1	1	0	1	1	0	NA	1	1	1	1	13	87	High
Kramer et al. (2017) [49]	1	1	1	1	(1,1) 1	1	1	1	1	1	0	NA	1	1	1	0	13	87	High
Kramer et al. (2021) [51]	1	1	1	1	(1,1) 1	1	1	0	1	1	1	NA	1	1	1	0	13	87	High
Kurtz et al. (2019) [32]	1	1	1	1	(1,1) 1	1	1	0	1	1	0	NA	1	1	1	0	12	80	High
Madsen et al. (2018) [52]	1	1	1	1	(1,1) 1	1	1	1	1	0	0	NA	1	0	1	0	11	73	Moderate
Martinent et al. (2018) [45]	1	1	1	1	(1,1) 1	1	1	1	1	1	0	1	1	1	1	1	15	94	High
Robertson et al. (2022) [43]	1	1	1	0	(1,1) 1	1	1	1	1	1	0	1	1	1	1	1	14	88	High
Sánchez-Muñoz et al. (2020) [39]	1	1	1	1	(1,1) 1	1	1	0	1	1	0	1	1	1	1	1	14	88	High
Sánchez-Pay et al. (2021) [41]	1	1	1	1	(1,1) 1	1	1	0	1	1	1	NA	1	1	1	0	13	87	High
Siener and Hohmann (2019) [56]	1	1	1	1	(1,1) 1	1	1	NA	1	1	0	1	1	1	1	1	14	93	High
Siener et al. (2021) [59]	1	1	1	1	(1,1) 1	1	1	0	1	1	0	1	1	1	1	1	14	88	High
Söğüt (2017) [33]	1	1	1	1	(1,1) 1	1	1	0	1	0	0	1	1	1	1	0	12	75	Moderate
Ulbricht et al. (2016) [42]	1	1	1	1	(1,1) 1	1	1	0	1	1	0	1	1	0	1	0	12	75	Moderate
Zhao et al. (2020) [60]	1	1	1	1	(1,1) 1	1	1	1	1	0	0	NA	1	1	1	1	13	87	High
Ziemann et al. (2011) [36]	1	1	1	0	(1,1) 1	1	1	0	1	0	0	1	0	0	1	0	9	56	Low

(1) Informative and balanced summary of what was done and what was found including the study’s design; (2) scientific background and rationale for the investigation is reported; (3) statement of clear, specific objectives and/or any pre-specified hypotheses is provided; (4) information on setting, locations, and relevant dates for data collection are provided. This must include information on sport, level of competition, data collection dates, and context to be scored as a ‘1’; (5a) characteristics of study participants (must include: age, sex, skill level, overall number) are provided; (5b) procedure of selecting athletes (e.g., eligibility criteria) and how participants were categorized for study purposes (e.g., expert and novice) is described; (6) clear definition of all outcome variables, sources of data and detailed description of assessment/measurement methods are provided; (7) clear description of all statistical methods, including those used to control for confounding, is provided; (8) how missing or incomplete data were handled is explained; (9) Main findings and results (e.g., percentage of participants being separated into their performance level correctly; participants whose performance was predicted correctly; percentage (e.g., variance) explained or predicted by the measured outcome variables) are reported; (10) statistical estimate(s) and precision values (e.g., 95% confidence interval) are provided for each group. A measure of effect size is provided (e.g., Cramer's V, phi coefficient, Cohen’s w); (11) sources of potential bias are taken into account; (12) post hoc comparisons between groups (e.g., expert vs novice) are provided when appropriate (i.e., overall test is significant); (13) a summary of key results with reference to study objectives is provided; (14) discussion of the study’s limitations is provided; (15) a cautious overall interpretation of results considering objectives and relevant evidence is provided; (16) discussion of the study results’ generalizability to similar or other contexts is provided. Numbers for the total are presented as rounded numbers

### Multidimensional Studies

Table 1 presents the included articles (*n* = 15) using a multidimensional approach. Twelve studies included measurements of two characteristics. Of these, six studies measured a combination of physiological and technical characteristics, and five studies focused on the combination of anthropometric and physiological characteristics. The combination of technical and tactical characteristics was considered within one study.

There was strong evidence that better physiological characteristics (i.e., aerobic fitness/endurance) and technical characteristics (i.e., serve and stroke velocity) are associated with or can explain higher performance in tennis [29–33]. These findings were confirmed in all studies using univariable [31, 33] and multivariable statistical approaches [29, 30, 32]. Limited evidence was found that better physiological characteristics (i.e., upper limb and lower limb angular velocities) are related to a higher performance level in table tennis using univariable statistics since only one study concerning this characteristic could be included for this sport [34].

Studies including anthropometrics and physiological characteristics revealed conflicting evidence for body composition and moderate evidence for physiological characteristics (i.e., aerobic capacity) as determinants for performance in tennis [35, 36]. Also, within these studies, better aerobic parameters appear to be associated with higher performance. Moreover, limited evidence was found for badminton, squash, and padel concerning anthropometrics and physiological determinants as only one article could be included for these characteristics per sport [37–39]. In general, a trend can be recognized that better physiological outcomes are related to a higher performance level. Such a trend was not visible regarding the anthropometric outcomes. All studies focusing on anthropometrics and physiological characteristics used univariable statistical approaches.

Finally for the studies investigating two characteristics, the combination of technical and tactical characteristics was evaluated in one study. As such, the level of evidence for these characteristics is limited. This study showed that higher-level players outperform lower-level players in technical and tactical skills in squash [40]. The findings in this study were based on a univariable statistical approach.

Three studies included measurements of three performance characteristics. Of these, two studies in tennis measured a combination of anthropometric, physiological, and technical characteristics [41, 42]. Again, a trend was found that performance is associated with anthropometric, physiological, and technical characteristics. Higher-level players tend to outscore lower-level players on various anthropometric, physiological, and technical outcomes. Based on the results of these two studies, the level of evidence must be classified as limited for these findings. Both studies included univariable statistics but one study only found a trend and no statistically significant differences between performance groups [41].

Another three-dimensional study was conducted in badminton including anthropometric, physiological, and psychological characteristics [43]. This study used a multivariable statistical approach to predict players’ performance level (i.e., elite, sub-elite, or novice). The combination of anthropometric, physiological, and psychological data classified 100% of the players correctly while 80% were cross-validated correctly. Since this was the only study in badminton evaluating these characteristics, the level of evidence is limited.

### Longitudinal Studies

Table 2 presents the included articles (*n* = 17) using a longitudinal approach. Three articles followed longitudinal and unidimensional approaches with one study assessing physiological characteristics in tennis [44], one study analyzing psychological characteristics in table tennis [45], and one study evaluating anthropometric characteristics in tennis [46]. All studies found statistically significant effects for the respective characteristics. That is, higher values for maximum oxygen uptake (VO_2_-max) in a cross-correlation analysis [44] and body mass index (BMI; assumed to be reflecting more muscle mass) in a polynomial regression model [46] are related to more success at the elite level in tennis. In table tennis, players on the international and national level showed higher values for motivation, coping skills, and stress tolerance compared to regional level players in an analysis of covariance (ANCOVA) [45]. Since there was only one study per characteristic, the level of evidence for all three of them is classified as limited [44–46].

Nine longitudinal studies included measurements of two characteristics. Most studies measured a combination of anthropometric and physiological characteristics (*n* = 6), of which five were conducted in tennis while one included badminton players. There was strong evidence that anthropometric (i.e., cross-sectional area of the quadriceps femoris muscle) and even more so physiological characteristics (i.e., lower body power [e.g., sprinting, jumping] and upper body power [e.g., medicine ball throwing]) were related to success and performance level in tennis [47–51]. However, it must be noted that there were some conflicting findings when evaluating characteristics in detail. For example, one study found relationships or differences for sprinting or jumping tests [48] while another did not [49]. As there was only one longitudinal study presenting results for physiological characteristics in badminton (i.e., jumping, badminton-specific speed, and endurance), this evidence was classified as limited [52]. All six studies used multilevel or multivariate analyses.

Two studies investigated a combination of physiological and technical characteristics. Both studies investigated whether perceptual-motor skills could predict future performance in competitive elite youth table tennis players using multilevel or multivariate analyses [53, 54]. While the assessments for technical characteristics (e.g., throwing a ball, aiming at target) showed statistically significant effects in both studies and thus represent moderate evidence, physiological characteristics (i.e., sprint, agility, jumping) showed effects only in one study and the evidence must be classified as conflicting accordingly. As above, it must be noted that there were more conflicting findings once characteristics were analyzed in deeper detail with respect to specific assessment and measurement methods. For example, one study found a positive effect for eye-hand coordination [53] while another did not [55]. Similarly, two studies found positive effects for sprinting in table tennis [54, 55] while two other studies did not find such effects [53, 56].

Finally for the longitudinal studies examining two characteristics, one study investigated a combination of anthropometric and technical characteristics in tennis using a multivariate analysis and found that future elite players were significantly taller and heavier compared to future competitive players. Also, ball speed and accuracy were significant predictors of current and future performance [57]. As this was only one study, the evidence was classified as limited.

Four longitudinal studies included measurements of three characteristics. Of these, three studies, two in tennis and one in table tennis, measured a combination of anthropometric, physiological, and technical characteristics [56, 58, 59] while one study combined anthropometric, physiological, and psychological characteristics in table tennis and badminton [60]. The first three studies found statistically significant effects for physiological (i.e., sprinting, jumping), and technical characteristics (e.g., ball throw) using a univariate as well as a multilevel analysis [56, 58, 59]. Conflicting findings were found for the predictive value of anthropometrics (i.e., height, weight) [56, 58, 59]. Accordingly, overall, there was moderate evidence for tennis and table tennis combined and limited evidence for each sport separately. In this context, it must be noted that anthropometric assessments were often used not as primary variables but rather as additional variables (i.e., to assess maturation) for the interpretation of, e.g., physiological or technical characteristics [36, 42, 43, 48–51, 55, 57, 58]. The last study with three characteristics evaluated the longitudinal development of anthropometric, physiological, and psychological characteristics over a 2-year period in male table tennis and badminton players using a discriminant analysis and multilayer perceptron neural network. The study found that all players improved in all aspects [60]. Accordingly, there was limited evidence that players get taller, heavier, fitter, and technically better over time.

Only one multidimensional and longitudinal study investigated four performance-related characteristics using correlation analysis. This study explored whether multidimensional profiling could be useful in predicting table tennis player’s current and future performance level one year later [55]. Statistically significant Spearman rank-order correlations were found for physiological (i.e., sprint) and a few psychological characteristics (e.g., work engagement, self-regulation). As there was only one study, the evidence level was classified as limited.

## Discussion

This systematic review aimed to provide an overview of empirical/data-driven multidimensional and/or longitudinal research in talent development within the field of racket sports. It intended to gain further insight into the outcomes of multidimensional and longitudinal approaches for talent identification and development in racket sports and provide directions for future talent research.

The findings show that multidimensional and longitudinal studies are being conducted in racket sports, especially in tennis and table tennis. However, despite the relatively high number of multidimensional and longitudinal studies (*n* = 32), it remains difficult to draw strong conclusions. This is mainly due to the lack of uniformity in various aspects of the studies. There are differences in the (1) operationalization of constructs (i.e., variables measured), (2) measurement instruments (i.e., how to measure these variables), (3) study designs, and (4) statistical approaches. Furthermore, despite the mostly moderate to high ratings for methodological quality, the study designs and statistical approaches that have been applied did not always seem to maximize the datasets’ added value for talent research. In approximately 50% (14/29) of the included multidimensional studies, a univariate analysis was chosen, where a multivariate analysis perhaps could have been more informative to further unravel the complexity of talent and its multidimensional nature. Although the chosen analyses were appropriate for the aims of the individual studies, optimally, statistical analyses should utilize the *potential of multidimensional data* and conduct multivariate analyses in talent research that comprise a set of performance characteristics. Consequently, characteristics are not only investigated in isolation but specifically in combination to reveal potential interaction effects. Such an approach offers the possibility to shed light on the so-called ‘compensation phenomenon’ [3, 61]. When players score poorly on certain performance characteristics, they can potentially compensate for this by well-developed other characteristics. This kind of information helps to better understand talent identification as well as developmental processes. In addition, statistical analyses should utilize the *potential of longitudinal data* and conduct not just cross-sectional analyses, but analyses that capture the performance characteristics on several points in time. Here, a focus on the difference in scores between those time points also appears valuable. That way, essential information can be revealed about improvement, stability or even decrement of a player’s performance characteristics in certain periods during their sports career [12].

Future research should aim to find best-practice assessments for various performance characteristics and use adequate (multivariate) statistical analyses to carefully interpret the results in detailed ways. A recent example for handling multidimensional data can be found in research reported by Robertson et al. (2022) who followed these steps in a best-practice manner [43]. The authors analyzed various variables regarding three different characteristics in three different sub-samples using a multivariate analysis of covariance (MANCOVA) and a discriminant analysis. Examples of best practice in longitudinal research can be found in studies applying multilevel modeling (see for an overview Elferink-Gemser et al. 2018 [4]). Multilevel modeling is an extension of multiple regression, which is appropriate for analyzing hierarchically structured data [62]. A two (or more) level hierarchy can be defined, with the repeated measurements (i.e., level-1) nested within the individual players (i.e., level-2). An advantage of using a multilevel regression modeling approach is that both the number of measurements and the temporal spacing of the measurements may vary between players [63]. This addresses one of the main challenges in longitudinal research, i.e., how to deal with incomplete datasets which are quite common in long-lasting research with humans. A multilevel model not only describes underlying population trends in a response (the fixed part of the model), but also models the variation around this mean response using the time of measurement and individual differences (the random part). In addition, researchers may need to use statistical analyses focusing on individual differences and development instead of group comparisons as world-class athletes are by definition outliers within statistical analyses.

While acknowledging the limitations in bringing together the multitude of studies in the current review, several relevant trends can still be observed. It became clear that almost all studies measured physiological characteristics in combination with either anthropometric, technical, or psychological characteristics. Anthropometric characteristics are frequently used for the interpretation of other outcomes. For example, an athlete’s maturation status (e.g., age at peak height velocity, APHV) can be determined based on anthropometric measures [64, 65]. Consequently, these measures provide information for the interpretation of other characteristics [48, 49, 51, 55, 57]. To illustrate, strong evidence was found that body height is related to serve speed in tennis [41, 66–69], while other studies found that serve speed was related to overall performance in tennis [32, 33, 42]. Together, this example shows how anthropometrics may be indirectly related to performance.

In some instances, the studies allowed for an insight into the individual racket sports in line with their task-specific demands. In tennis, strong evidence was found for physiological characteristics in both multidimensional [29–33, 35, 36, 41, 42] and longitudinal studies [44, 49, 59]. Physiological characteristics related to motor coordination, sprint, strength, and endurance were reported to be beneficial for progressing through TID programs [58] and to increase a player’s chances of achieving expert performance. In badminton, more advanced players performed better on motor fitness [37], explosive power, flexibility, endurance, and speed [43]. When investigated longitudinally, speed and endurance improved with age, and youth players (U19) reached comparable speed levels to those of world-class players [52]. In table tennis, more advanced players performed higher joint torques at higher racket speeds [34]. Also, current performance and performance progression were found to be related to sprint speed in youth table tennis players [55]. For squash and padel, limited evidence was found for the relationship between physiological characteristics and performance given that only two studies were conducted [38, 39].

Regarding technical characteristics, limited evidence was found for the different types of multidimensional and longitudinal studies separately, while moderate evidence was found when all were combined. Moderate to high correlations were found between technical characteristics and performance measures [29, 32], and technical skills were able to discriminate between performance levels in tennis [29]. This is in accordance with a previous systematic review of research on technical and tactical skills in tennis which found technical skills (e.g., ball velocity and ball accuracy) to be discriminative [20]. Also, differences in technical characteristics were found between performance levels in squash [40]. In both tennis and table tennis, technical characteristics were able to predict future performance [20, 53, 54, 56, 59] and were considered important for progression through several stages of a TID program [58]. Similarly, this was shown for, among others, perceptual abilities and coordinative skills in a previous systematic review [19]. In most sports, technical and tactical characteristics are very strongly connected. Technique often plays a functional role in executing a tactical decision to reach a certain goal [20]. However, only a single study investigated tactical characteristics using a multidimensional and/or longitudinal approach. Therefore, only limited evidence was found for the relationship between tactical characteristics and performance [40]. It is important to note that a previous review found many cross-sectional and unidimensional studies investigating (technical and) tactical or perceptual-cognitive skills in racket sports [20]. This fact further emphasizes the need for longitudinal research.

Although only limited evidence was found for psychological characteristics in the different types of multidimensional and longitudinal studies separately, it must be mentioned that several articles did find that psychological characteristics were related to (future) performance [43, 45, 55]. These studies used different questionnaires (e.g., Psychological Characteristics of Developing Excellence Questionnaire, version 2 [PCDEQ2], or Sport Motivation Scale [SMS]) to assess various psychological concepts and skills. For example, this included intrinsic and extrinsic motivation, self-regulation, coping skills, and mental toughness. These findings are similar to a previous systematic review, finding moderate evidence for the assessment of mental and goal management skills in racket sport players [19].

Some limitations of this systematic review must be acknowledged. First of all, this review focused only on one part of the GSTM [13] as it isolated the athlete’s individual characteristics and did not include valuable information beyond the individual (i.e., task and environmental characteristics). For example, sociodemographic characteristics, such as family support or coaching situation could have an influence on whether the athlete has the possibility to play at an elite level [6, 13, 70–72]. The inclusion of this information is recommendable for a better understanding of the complexity of talent identification and athlete development. Secondly, some measures were difficult to categorize into a single individual characteristic because of the complex interaction between characteristics. To illustrate, eye-hand reaction time consists of both a psychological/cognitive and a technical-motor component [73] and could therefore be categorized as a technical or psychological characteristic. Depending on this categorization decision, a study could have been considered unidimensional instead of multidimensional and consequently been excluded for this reason. Thus, it is important for all researchers in the field to create and report their studies using generic terminology and categorization so that all relevant information from the literature can be captured and combined. This must include specifically the study’s design, statistical approach, and participants’ age and performance level. Thirdly, the findings of this review may have been influenced by a publication bias. Although the most commonly used databases for sport settings were used [23], a wider search among more databases including not only English-language studies and other study designs and/or grey literature might yield new insights. For example, no studies on coaches’ perspectives were included, even though they can be of added value when identifying important individual characteristics and their relationship to performance [74–76]. Moreover, publication bias within the empirical/data-driven studies might be due to favoring statistically significant and positive results over null or negative/conflicting results. Particularly given the comparison and combination of results of different studies and seeing partially conflicting findings (e.g., for sprinting), this may be relevant. Thus, all researchers should strive to improve publication procedures using new and more transparent approaches (e.g., pre-registration). Fourthly, although racket sports share certain task similarities, there are still differences in demands between them, e.g., physiologically [76, 77], and the settings in which the measurements take place (i.e., a laboratory versus ecological-valid context, e.g., in a competitive setting) must be considered when interpreting and transferring results. Consequently, generalization of findings from one racket sport to another, as well as from certain (assessment) settings to others, must be done with caution.

## Conclusions

In conclusion, this systematic review provides an overview of talent research using multidimensional and/or longitudinal approaches within racket sports. Despite the apparent challenges of bringing together the variety of current multidimensional and longitudinal studies, this review revealed pieces of relevant information on which individual performance-related characteristics could help explain performance outcomes in young talented racket sport players. Depending on the specific sport of interest, the current literature provides both practitioners and researchers some guidance on what characteristics to include in their decision-making processes in TID contexts. Future research should conduct more multidimensional and longitudinal studies combining various individual and also environmental characteristics. Characteristics should be assessed using (standardized) best-practice methods to allow for better comparisons and combinations of studies. Also, data should be analyzed using adequate (multivariate) analyses to effectively take advantage of multidimensional and longitudinal approaches’ added value. When presenting their findings, researchers should use generic terminology and extensively describe the study’s methodological approach and sample. All in all, this helps to continue unraveling the concepts and processes underlying successful talent identification and development in (racket) sports.

### Supplementary Information

**Additional file 1**. **Table S1**: Overview of the distribution of individual characteristics in the included multidimensional studies.

## Data Availability

The datasets generated during and/or analyzed during the current study are available from the corresponding author on reasonable request.

## Abbreviations

TID: Talent identification and development
GSTM: Groninger Sports and Talent Model
PRISMA: Preferred Reporting Items for Systematic Reviews and Meta-Analyses
STROBE: Strengthening the reporting of observational studies in epidemiology
NA: Not applicable
BMI: Body mass index
ANCOVA: Analysis of covariance
MANCOVA: Multivariate analysis of covariance
