# Supplementary material for: Multidimensional and Longitudinal Approaches in Talent Identification and Development in Racket Sports: A Systematic Review
Source: Sports Med Open. 2024 Jan 7;10:4. doi: 10.1186/s40798-023-00669-2 (PMC10772031; doi:10.1186/s40798-023-00669-2)
Supplement: Supplementary file 1 — Additional file 1. Table S1: Overview of the distribution of individual characteristics in the included multidimensional studies. [file 40798_2023_669_MOESM1_ESM.docx]

**Supplementary material to:**

For Sports Medicine

**Multidimensional and Longitudinal Approaches in Talent Identification and Development in Racket Sports: A Systematic Review**

Sebastiaan B. Nijenhuis^1#^,

Till Koopmann^2#^,

Jesper Mulder^1^,

Marije T. Elferink-Gemser^1^,

Irene R. Faber^2,3^

^1^Department of Human Movement Sciences, University of Groningen, Groningen, The Netherlands

^2^Institute of Sport Science, Carl von Ossietzky University Oldenburg, Oldenburg, Germany

^3^Research Centre Human Movement and Education, Windesheim University of Applied Sciences, Zwolle, The Netherlands

ORCIDs:

Till Koopmann [https://orcid.org/0000-0002-3985-4084](NULL)

Irene Faber [https://orcid.org/0000-0002-4994-0453](NULL)

Marije Elferink-Gemser https://orcid.org/0000-0003-2555-4782

^#^These authors share first authorship

**Corresponding author:*

Irene Faber

Institute of Sport Science, Carl von Ossietzky University Oldenburg,

Ammerländer Heerstraße 114-118

26129 Oldenburg

Phone +31 6 200 30 686

E-mail: [i.r.faber@windesheim.nl](NULL)

Running head: Multidimensional and longitudinal talent approaches in racket sports

## Supplementary table

Table S1. Overview of the distribution of individual characteristics in the included multidimensional studies

| **First author (year)** | **Anthropometrics** | **Physiological** | **Technical** | **Tactical** | **Psychological** |
| --- | --- | --- | --- | --- | --- |
| Abdullahi (2017) | X | X |  |  |  |
| Baiget (2014) |  | X | X |  |  |
| Baiget (2016) |  | X | X |  |  |
| Banzer (2008) | X |  |  |  |  |
| Brechbuhl (2018) |  | X | X |  |  |
| Catalán-Eslava (2018) |  |  | X | X |  |
| Chapelle (2022) | X | X | X |  |  |
| Chen (2022) |  | X | X |  |  |
| Doherty (2018) | X | X | X |  | X |
| Faber (2016) |  | X | X |  |  |
| Faber (2017) |  | X | X |  |  |
| Filipčič (2015) | X | X |  |  |  |
| Gale-Watts (2016) |  | X |  |  |  |
| James (2022) | X | X |  |  |  |
| Kanehisa (2006) | X | X |  |  |  |
| Kolman (2021) | X |  | X |  |  |
| Kramer (2016a) | X | X |  |  |  |
| Kramer (2016b) | X | X |  |  |  |
| Kramer (2017) | X | X |  |  |  |
| Kramer (2021) | X | X |  |  |  |
| Kurtz (2019) |  | X | X |  |  |
| Madsen (2018) | X | X |  |  |  |
| Martinent (2018) |  |  |  |  | X |
| Robertson (2022) | X | X |  |  | X |
| Sánchez-Muñoz (2020) | X | X |  |  |  |
| Sánchez-Pay (2021) | X | X | X |  |  |
| Siener (2019) | X | X | X |  |  |
| Siener (2021) | X | X | X |  |  |
| Söğüt (2017) |  | X | X |  |  |
| Ulbricht (2016) | X | X | X |  |  |
| Zhao (2020) | X | X |  |  | X |
| Ziemann (2011) | X | X |  |  | X |
| **Total (*n =* 32)** | **21** | **28** | **16** | **1** | **4** |
| *^1^The technical category covered both technical skills and (perceptuo-)motor skills.  ^2^The psychological category covered both psychological and cognitive skills.* | | | | | |
